# Longitudinal Changes in Systemic Total Oxidant Status Following High-Intensity Focused Ultrasound for Facial Rejuvenation: A Prospective 6-Month Controlled Pilot Study

**DOI:** 10.3390/s26185941

**Published:** 2026-09-19

**Authors:** Foteini Biskanaki, Vasilis Papadopoulos, Ioanna A. Anastasiou, Nikolaos Tentolouris, Ilias Velissariou, Sofia Georgia Pantazi, George Skouras, Dimitrios Chaniotis, Fragiskos Chaniotis, Effie G. Papageorgiou, Athanasia Varvaresou, Evaggelia Protopapa, Andreas C. Lazaris

**Affiliations:** 1 First Department of Pathology, School of Medicine, The National and Kapodistrian University of Athens, 11527 Athens, Greece; eliasvelissariou@gmail.com (I.V.); sophiepantazi12@gmail.com (S.G.P.); alazaris@med.uoa.gr (A.C.L.); 2 Department of Biomedical Sciences, School of Health and Care Sciences, University of West Attica, 12243 Athens, Greece; dchaniotis@uniwa.gr (D.C.); fchaniotis@uniwa.gr (F.C.); efipapag@uniwa.gr (E.G.P.); avarvares@uniwa.gr (A.V.); evi.protopapas@gmail.com (E.P.); 3 Diabetes Center, First Department of Propaedeutic Internal Medicine, School of Medicine, The National and Kapodistrian University of Athens, 11527 Athens, Greece; anastasiouiwanna@gmail.com (I.A.A.); ntentol@med.uoa.gr (N.T.); 4 Department of Pharmacology, The National and Kapodistrian University of Athens, 11527 Athens, Greece; 5 Department of Plastic Surgery, SkourasMed Clinic, 10673 Athens, Greece; giorgos_skouras@yahoo.gr

**Keywords:** high-intensity focused ultrasound (HIFU), oxidative stress, total oxidant status, ELISA, systemic biomarker monitoring, facial rejuvenation, longitudinal assessment

## Abstract

**Highlights:**

**What are the main findings?**

- Serum TOS levels were significantly lower in the HIFU group than in controls at 3 and 6 months.
- The lowest TOS values occurred at 3 months, followed by a modest increase at 6 months.
- A single HIFU session did not induce a sustained systemic oxidative burden in healthy adults.

**What are the implications of the main findings?**

- Longitudinal ELISA-based monitoring provides an objective approach for systemic redox shifts.
- Dermal thermal coagulation by HIFU triggers a favorable, long-term systemic redox regulation.
- The absence of prolonged oxidative stress confirms the excellent safety profile of HIFU.

**Abstract:**

High-intensity focused ultrasound (HIFU) is widely used for non-invasive facial and neck rejuvenation; however, its longer-term systemic biochemical effects remain insufficiently characterized. This prospective controlled pilot study investigated longitudinal changes in systemic oxidative status at baseline and 3 and 6 months following a single HIFU treatment. Thirty healthy adults were enrolled, including 25 participants who underwent facial and neck HIFU and 5 untreated participants serving as a comparison group. Serum total oxidant status (TOS) was quantified using an enzyme-linked immunosorbent assay (ELISA)-based method, and longitudinal changes were assessed using mixed-effects modeling. Baseline TOS did not differ significantly between groups (19.54 ± 1.49 vs. 19.78 ± 1.81 μmol/L; *p* = 0.794). A significant group × time interaction was observed (Wald χ^2^(2) = 21.78, *p* < 0.001). Within the HIFU group, TOS decreased at 3 months (17.31 ± 1.51 μmol/L; Bonferroni-adjusted *p* < 0.001) and remained below baseline at 6 months (18.34 ± 0.95 μmol/L; *p* < 0.001), despite a partial increase between 3 and 6 months (*p* = 0.002). Three participants (12.0%) reported mild, transient deep-tissue tenderness; no serious adverse events occurred. Overall, HIFU was associated with a distinct longitudinal TOS pattern, supporting further investigation of redox and tissue-remodeling biomarkers.

## 1. Introduction

High-intensity focused ultrasound (HIFU) is increasingly used as a non-invasive modality for facial and neck rejuvenation. By delivering focused acoustic energy to predefined tissue depths, HIFU creates localized thermal coagulation zones while largely preserving the epidermal surface. This controlled thermal stimulation initiates collagen contraction and subsequent tissue-remodeling processes, including fibroblast activation, neocollagenesis, elastin remodeling, and extracellular-matrix reorganization [1,2]. Clinical studies have reported improvements in facial and neck laxity, wrinkles, skin elasticity, and contour, with treatment effects developing progressively during the months following treatment [3,4,5,6].

Although the clinical efficacy and local safety profile of aesthetic HIFU have been increasingly investigated, its longer-term systemic biochemical effects remain insufficiently characterized. Controlled tissue stimulation may involve transient oxidative and inflammatory signaling as part of the biological response to tissue injury and repair. Reactive oxygen species (ROS), when generated at controlled levels, participate in cellular signaling and adaptive responses, whereas sustained oxidant production exceeding antioxidant capacity may contribute to cellular damage and oxidative stress [7,8,9,10]. Distinguishing a transient redox response from persistent systemic oxidative burden is therefore relevant when evaluating an energy-based intervention designed to induce controlled tissue remodeling.

Total oxidant status (TOS) provides an integrated estimate of the overall oxidant burden within a biological sample rather than quantifying individual oxidant species separately [11]. Serum TOS may therefore provide a useful quantitative measure for longitudinal assessment of systemic redox changes following HIFU. However, previous investigations of aesthetic HIFU have primarily focused on clinical outcomes, tissue remodeling, treatment tolerability, and adverse effects [3,4,5,6,12,13], while longitudinal human data on systemic oxidant status during the months following treatment remain limited.

In a previous pilot investigation, our research group evaluated the acute systemic oxidative response following facial and neck HIFU treatment [14]. These findings highlighted the need to extend biomarker assessment beyond the immediate post-treatment period and to determine how systemic oxidant status evolves during the subsequent months.

Accordingly, the present prospective controlled pilot study aimed to evaluate serum TOS at baseline and at 3 and 6 months following a single facial and neck HIFU session and to compare its longitudinal trajectory with that observed in an untreated comparison group. We hypothesized that a single HIFU treatment would not be associated with sustained systemic oxidative burden during the 6-month follow-up period and that the longitudinal TOS profile would differ between HIFU-treated and untreated participants.

In this context, the study focuses not on the development of a new sensor device but on the longitudinal application of a standardized quantitative biochemical measurement approach to characterize systemic redox responses following HIFU.

## 2. Materials and Methods

### 2.1. Study Design

This prospective, controlled, non-randomized pilot study was conducted at the First Department of Pathology, School of Medicine, National and Kapodistrian University of Athens, Athens, Greece, between August 2025 and April 2026. Participant enrollment and HIFU treatment sessions were conducted between August and October 2025, while the final follow-up assessments and blood collections were completed between March and April 2026.

Participants were allocated to either the HIFU intervention group or the untreated comparison group according to their treatment preference; therefore, group allocation was not randomized. Laboratory personnel responsible for serum processing and biochemical analysis were blinded to group allocation and sampling timepoint to minimize analytical bias.

The present study focused on the longitudinal evaluation of serum total oxidant status at baseline and at the 3- and 6-month follow-up assessments. Immediate post-treatment measurements were not included or reanalyzed in the present manuscript, as the acute systemic biochemical response to HIFU has been reported separately in a previous publication [14].

The present study included 30 participants, comprising 25 participants in the HIFU group and 5 untreated participants in the comparison group, a sample size consistent with established methodological guidelines for exploratory pilot investigations [15,16,17]. Fourteen of the 25 HIFU-treated participants had also participated in our previously published acute-response investigation, which included 14 HIFU-treated participants and 5 controls. The present study extended the longitudinal follow-up of these HIFU-treated participants to 3 and 6 months and expanded the HIFU cohort by enrolling 11 additional participants.

For participants who had been included in the previous publication, baseline measurements were used as the longitudinal reference for the present analysis, whereas the previously reported immediate post-treatment measurements were not presented as new findings. All 3- and 6-month follow-up measurements analyzed in the present study were previously unpublished.

**Figure 1:**
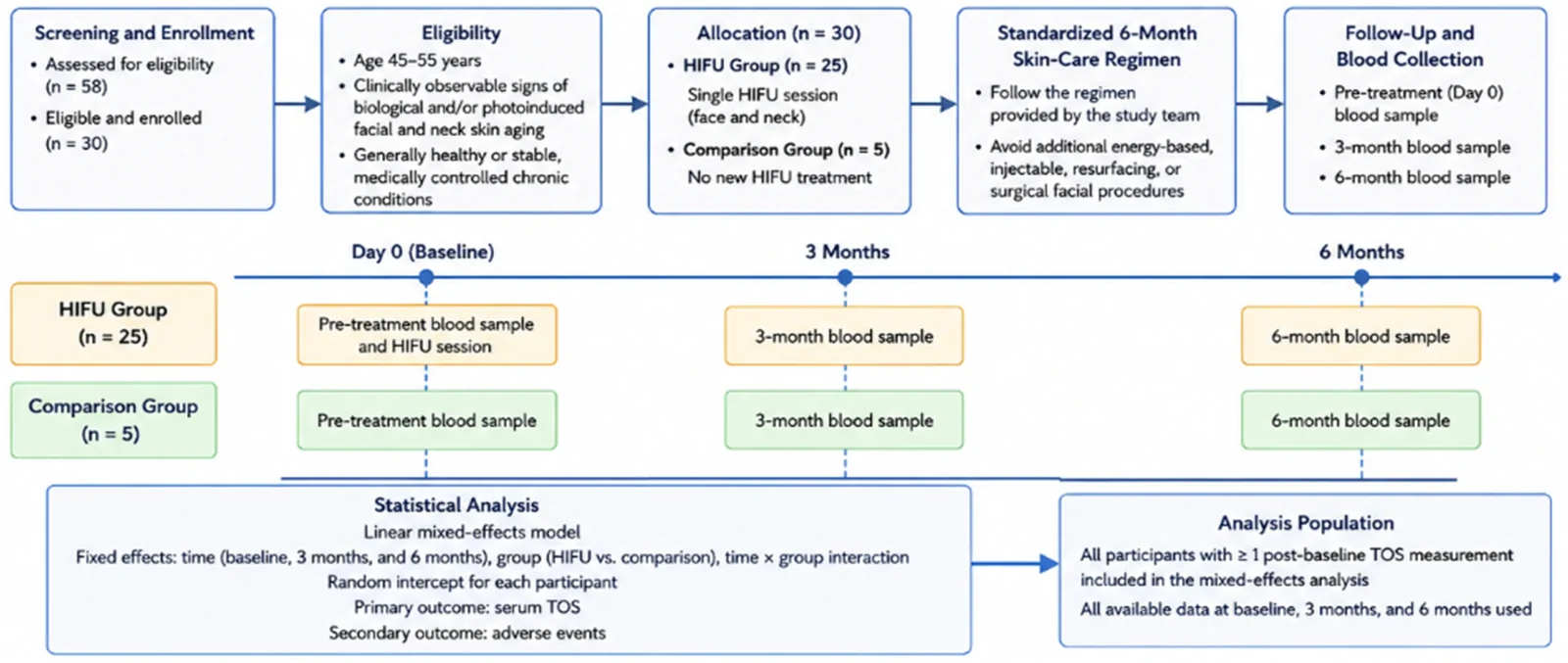
Flowchart of the study design, participant allocation, follow-up assessments, and serum total oxidant status (TOS) measurements. A total of 30 participants were included, comprising 25 participants in the HIFU group and 5 participants in the comparison group. Participants in the HIFU group underwent a single facial and neck HIFU treatment session, whereas the comparison group received no new HIFU treatment during the study period. Venous blood samples for serum TOS assessment were collected at baseline (pre-treatment), 3 months, and 6 months. The primary outcome was the longitudinal change in serum TOS, and the secondary outcome was treatment safety. Fourteen HIFU-treated participants had participated in the previously published acute-response study; 11 additional HIFU-treated participants were newly enrolled. All 3- and 6-month follow-up data presented in the current study were previously unpublished.

The overall study workflow, participant allocation, cohort overlap with the previous acute-response investigation, follow-up schedule, blood-sampling timepoints, and biomarker-assessment procedures are summarized in Figure 1.

### 2.2. Participants and Eligibility Criteria

A total of 30 adults aged 45–55 years were included in the study, comprising 28 females and 2 males. Twenty-five participants were allocated to the HIFU intervention group and five to the untreated comparison group.

Eligible participants were adults with clinically observable signs of biological and/or photo-induced facial and neck skin aging who were generally healthy or had stable, medically controlled chronic conditions. Participants were required to attend all scheduled blood collections, follow the standardized 6-month skincare regimen, and avoid additional energy-based, injectable, resurfacing, or surgical facial procedures during follow-up. They were instructed to refrain from smoking for at least 2 h and from vigorous physical exercise for at least 24 h before each blood collection.

Exclusion criteria included pregnancy or breastfeeding; active skin infection, inflammatory acne, or inflammatory dermatosis in the treatment area; uncontrolled or clinically significant systemic disease; immunosuppression; implanted electronic devices or metal implants within or near the treatment area; injectable facial treatment within the preceding 6 months; surgical facelift within the preceding 12 months; use of topical agents causing clinically relevant irritation; and any contraindication to HIFU treatment.

Previous HIFU exposure was recorded but was not considered an exclusion criterion.

### 2.3. Ethical Considerations and Informed Consent

The study was conducted in accordance with the Declaration of Helsinki and within the framework of an approved postdoctoral research protocol at the School of Medicine, National and Kapodistrian University of Athens. The research protocol received approval from the Bioethics and Ethics Committee of the Medical School prior to participant enrollment (Approval No. 1072/30 June 2025). All participants provided written informed consent before enrollment. The study was not separately registered in a public clinical-trial registry.

### 2.4. HIFU Treatment Protocol

Participants in the intervention group underwent a single high-intensity focused ultrasound (HIFU) treatment session targeting the face and neck. All procedures were performed by the first author, who had more than 8 years of clinical and academic experience in the aesthetic application of energy-based technologies.

Treatment was performed using a commercially available, CE-marked HIFU system intended for aesthetic facial and neck applications. Transducers operating at frequencies of 2–3 MHz were selected according to the anatomical region and intended focal depth. Focal depths of 3.0–4.5 mm were used for the neck and cheeks, whereas depths of 1.5–3.0 mm were used for the periorbital region and forehead.

Treatment parameters were individualized within predefined ranges according to the anatomical region, local tissue characteristics, and participant tolerance. Accordingly, a single uniform setting was not applied across the entire face and neck or across all participants, and different settings could be used within the same participant for the neck, cheeks, periorbital region, and forehead. Device-displayed energy settings ranged from 0.6 to 1.2 J. The predefined anatomical treatment sequence was neck, cheeks, periorbital region, and forehead. A total of 380–480 shots were delivered per participant across the face and neck, with an overall treatment duration of approximately 40–60 min.

The treatment head delivered each shot through a scanning pattern of five treatment lines, generating a total of 40–50 focal points per shot. The device did not provide automated shot-location tracking. Before treatment, the facial and neck treatment areas were systematically mapped directly on the skin using a white cosmetic pencil, with markings corresponding to the treatment-head footprint. Each treatment application was delivered with the treatment head held stationary within the predefined marked field before being repositioned to the next marked treatment site. This pre-treatment mapping provided a visual guide for systematic coverage of the predefined anatomical treatment zones.

The device did not provide real-time tissue-temperature feedback. Treatment delivery and safety were therefore guided by the predefined anatomical treatment plan, device-displayed parameters, anatomical characteristics of each treatment region, and continuous clinical assessment of participant tolerance.

Pain intensity was assessed at 10-min intervals using an 11-point numerical rating scale ranging from 0, indicating no pain, to 10, indicating the worst imaginable pain. Brief treatment pauses were permitted when required according to participant comfort.

All procedures were performed in accordance with the manufacturer’s instructions for use and established safety precautions. Before treatment, a standard water-based ultrasound coupling gel, commonly used in diagnostic ultrasonography, was applied to the treatment areas to facilitate acoustic transmission and maintain consistent contact between the transducer and the skin. No additional energy-based, injectable, or minimally invasive aesthetic treatment was administered as part of the HIFU intervention.

### 2.5. Standardized Post-Treatment and Home-Care Regimen

The post-treatment and home-care regimen was predefined specifically for the present study to standardize topical skincare exposure across participants and was not intended to represent an institutional or hospital standard-of-care protocol. The regimen was also not evaluated as an independent therapeutic intervention.

Immediately after completion of the HIFU procedure, Pure Mastic Serum was applied topically to the treated areas of the face and neck, followed by Intense Renewal Total Face Cream and Protect and Hydrate SPF 30 sunscreen, all manufactured by SkourasMed (Athens, Greece).

To standardize topical skin-care exposure throughout the follow-up period, participants in both the HIFU and comparison groups were provided with the same home-care products manufactured by SkourasMed (Athens, Greece). The standardized regimen was followed for 6 months and consisted of the following:

- Morning regimen: Pure Mastic Serum, followed by Intense Renewal Total Face Cream and Protect and Hydrate SPF 30 sunscreen.
- Evening regimen: Vitamin C Antioxidant Serum, followed by Intense Renewal Total Face Cream.

The Vitamin C serum was included as a component of the predefined standardized home-care regimen applied equally to both study groups and was not evaluated as an independent antioxidant intervention.

Participants were instructed to apply the products in the specified sequence every day throughout the 6-month study period. Sunscreen reapplication was recommended according to the duration and intensity of sun exposure. They were instructed not to introduce additional active cosmetic products, topical irritants, or energy-based, injectable, resurfacing, or other facial rejuvenation procedures during the follow-up period. Participant adherence to the standardized home-care regimen was assessed at both the 3- and 6-month follow-up visits using a self-reported numerical rating scale ranging from 1 to 10, with higher scores indicating greater adherence. The mean self-reported adherence score across the two follow-up assessments was 8/10, indicating generally high reported compliance with the prescribed regimen.

The same standardized skincare regimen was provided to both study groups to minimize differences in topical product exposure between groups. Because both groups received identical background skincare, the divergent longitudinal TOS trajectories observed during follow-up are less likely to be attributable solely to differences in topical skincare exposure. Nevertheless, the contribution of individual skincare ingredients to systemic TOS cannot be completely excluded because the present study was not designed to evaluate the independent effects of the skincare regimen.

### 2.6. Blood Collection and Serum Processing

For the present longitudinal analysis, venous blood samples were collected before treatment (baseline) and at 3 and 6 months after the HIFU treatment. Participants in the comparison group underwent blood collection at the corresponding time points following enrollment. Baseline, immediate post-treatment, and 1 h post-treatment measurements were not included or reanalyzed in the present study.

Blood samples were collected into 4.0 mL serum separator tubes (Vacutainer SST Lab Tubes; BD, Franklin Lakes, NJ, USA). The tubes were gently inverted five times to ensure adequate mixing and were subsequently allowed to clot for 30–60 min at room temperature. Samples were centrifuged at 2000× *g* for 15 min at either room temperature or 4 °C, according to the standardized laboratory protocol used in the previously published acute-response investigation [14].

Following centrifugation, the separated serum was transferred into clean, appropriately labeled tubes and stored at −80 °C within 30 min of centrifugation until biochemical analysis. All samples were processed according to the same standardized pre-analytical protocol to minimize variability associated with sample handling and storage.

### 2.7. Total Oxidant Status Assay and Analytical Quality Control

Serum total oxidant status (TOS) was quantified using a commercially available microplate-based colorimetric assay kit (Total Oxidant Status (TOS) Colorimetric Assay Kit, EEA027, Thermo Fisher Scientific, Waltham, MA, USA), in accordance with the manufacturer’s instructions and the analytical methodology previously described by our research group [14].

All serum samples were analyzed in duplicate. TOS concentrations were calculated from multi-point standard curves generated within the same analytical run, expressed in μmol/L, and recorded to two decimal places according to the numerical output of the analytical procedure. The second decimal place should not be interpreted as implying additional biological precision.

Each analytical run included reagent blanks, calibration standards, and low-, medium-, and high-concentration quality-control samples. Duplicate coefficients of variation were calculated to assess within-run precision, while between-run quality-control performance was monitored throughout the analytical process. The limits of detection and quantification, linear analytical range, and intra- and inter-assay coefficients of variation were documented for the assay lot used.

The same analytical procedures, quality-control criteria, and calculation methods were applied to samples from both study groups at all sampling time points to ensure consistency, reproducibility, and comparability of the measurements.

### 2.8. Study Outcomes

The primary outcome was the longitudinal change in serum total oxidant status (TOS) across the pretreatment baseline, 3-month, and 6-month assessments following a single HIFU treatment. Serum TOS trajectories in the HIFU group were also compared with those observed in the untreated comparison group over the corresponding follow-up period. TOS was the only circulating systemic biomarker analyzed in the present study; no additional systemic inflammatory or wound-healing biomarkers were assessed. The study was not designed to evaluate the technical performance or clinical efficacy of a specific HIFU platform; its primary objective was to characterize longitudinal systemic TOS changes following the applied HIFU intervention.

Standardized clinical photography and digital facial imaging were performed using the MEICET Pro-D skin analyzer (MEICET, Guangzhou, China) as part of the broader longitudinal research protocol for standardized clinical documentation. However, dermoanalyzer-derived parameters and photographic data were not designated as outcomes of the present biomarker-focused analysis and were therefore not included in the current statistical analysis or Results section.

The secondary outcome was treatment safety, assessed through systematic recording of participant-reported and investigator-observed adverse events throughout the 6-month follow-up period. Adverse events were characterized according to type, severity, duration, and potential relationship to the HIFU procedure.

Immediate post-treatment TOS measurements were not included or reanalyzed in the present study, as these acute-response data have been reported previously [14].

### 2.9. Statistical Analysis

Continuous variables are presented as mean ± standard deviation (SD) and categorical variables as frequencies and percentages. Distributional and model assumptions were assessed using graphical inspection, the Shapiro–Wilk test, and residual diagnostics.

Longitudinal changes in serum total oxidant status (TOS) were analyzed using a linear mixed-effects model with fixed effects for time (baseline, 3 months, and 6 months), study group (HIFU versus comparison), and the group × time interaction. A participant-specific random intercept was included to account for repeated measurements within individuals [18], and model parameters were estimated using restricted maximum likelihood.

Prespecified within-group and between-group comparisons were derived from estimated marginal means and adjusted using the Bonferroni method. Hedges’ g was used for between-group effect sizes and Cohen’s dz for within-group paired comparisons. All tests were two-sided, with statistical significance set at *p* < 0.05. Analyses were performed using IBM SPSS Statistics for Windows, version 26.0 (IBM Corp., Armonk, NY, USA).

### 2.10. Pilot Sample-Size Rationale

No formal a priori power calculation was performed because the study was designed as an exploratory pilot investigation. The sample was based on participant availability, feasibility of repeated blood collection, and the objective of obtaining preliminary estimates of the magnitude of and variability in longitudinal TOS measurements.

The study was not designed or powered as a definitive confirmatory trial. Accordingly, emphasis was placed on effect-size estimates, confidence intervals, feasibility, and the direction and consistency of the observed biomarker patterns rather than on *p*-values alone [15,16,17]. The estimates obtained from this pilot cohort may inform the design and sample-size calculation of a subsequent adequately powered, randomized investigation.

## 3. Results

### 3.1. Participant Characteristics

All 30 enrolled participants completed the 6-month follow-up and were included in the final analysis. The study population comprised 28 women and 2 men, with 25 participants in the HIFU group and 5 participants in the untreated comparison group. Complete serum total oxidant status (TOS) measurements were available for all participants at baseline and at the 3- and 6-month follow-up assessments.

Participants were generally healthy or had stable, medically controlled chronic conditions. None had an acute dermatological disorder, uncontrolled systemic disease, or any other condition contraindicating study participation. Baseline demographic, lifestyle, and clinical characteristics of the study population are presented in Table 1.

### 3.2. Longitudinal Changes in Serum Total Oxidant Status

Complete serum TOS data were available for all 30 participants at baseline and at the 3- and 6-month follow-up assessments. Observed TOS values and longitudinal changes for both study groups are summarized in Table 2.

The linear mixed-effects model demonstrated a significant group × time interaction (Wald χ^2^(2) = 21.78, *p* < 0.001), indicating that the longitudinal TOS trajectory differed between the HIFU and comparison groups.

There was no significant between-group difference in TOS at baseline (*p* = 0.794), whereas TOS was significantly lower in the HIFU group than in the comparison group at both 3 and 6 months (both Bonferroni-adjusted *p* < 0.001). Within the HIFU group, TOS decreased significantly from baseline to 3 months and remained significantly below baseline at 6 months (both Bonferroni-adjusted *p* < 0.001), despite a significant partial increase between 3 and 6 months (Bonferroni-adjusted *p* = 0.002). In the comparison group, the increase from baseline to 6 months was statistically significant (Bonferroni-adjusted *p* = 0.022), whereas the baseline-to-3-month and 3-to-6-month changes were not significant after Bonferroni correction.

Supplementary within-group analyses supported the mixed-model findings. In the HIFU group, the baseline-to-3-month change was normally distributed (Shapiro–Wilk W = 0.939, *p* = 0.140) and was associated with a large reduction in TOS (paired *t*-test: *t*(24) = −6.51, *p* < 0.001; Cohen’s dz = −1.30). As the baseline-to-6-month and 3-to-6-month paired differences deviated from normality, Wilcoxon signed-rank tests were used, confirming significantly lower TOS at 6 months compared with baseline (W = 17, *p* < 0.001) and significantly higher TOS at 6 months compared with 3 months (W = 57, *p* = 0.005).

### 3.3. Exploratory Participant-Level Patterns and Distribution of TOS Values

Given the limited sample size, the unequal allocation between the HIFU and comparison groups, and the small number of participants within several demographic and lifestyle categories, formal confirmatory subgroup analyses were not considered sufficiently powered. Exploratory assessment did not reveal consistent descriptive patterns suggesting that the longitudinal changes in serum total oxidant status were systematically associated with age, menopausal status, smoking, alcohol consumption, physical activity, self-reported stress, sun exposure, sunscreen use, medication history, daily water intake, or previous HIFU treatment. These observations should be regarded as hypothesis-generating and should not be interpreted as evidence of the absence of subgroup effects.

**Figure 2:**
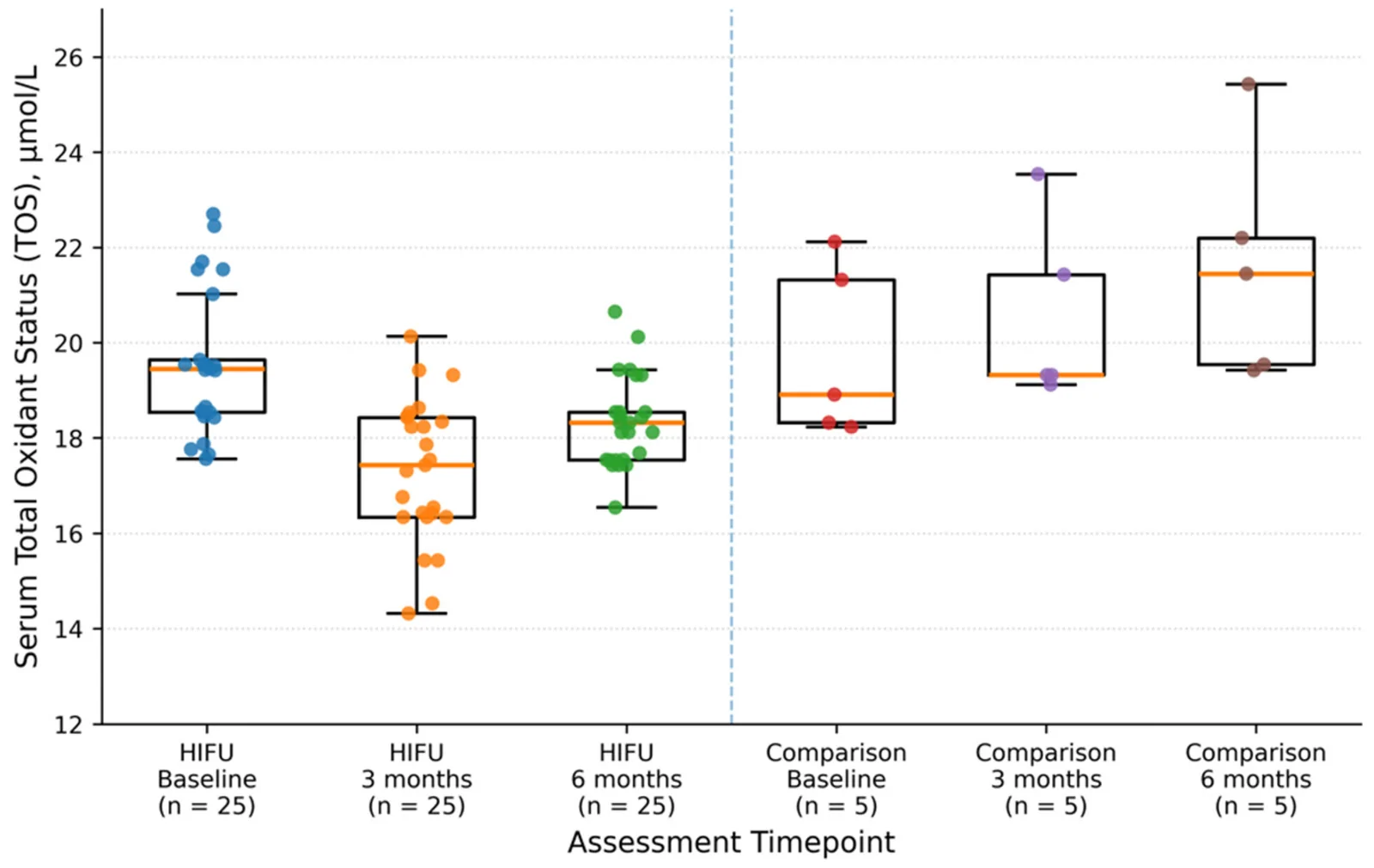
Distribution of serum total oxidant status in the HIFU and comparison groups at baseline and at the 3- and 6-month follow-up assessments. Boxplots represent the median and interquartile range, with individual participant values overlaid as points. In the HIFU group, serum TOS values demonstrated a downward shift at 3 months, followed by a partial increase at 6 months. In contrast, the comparison group demonstrated a progressive upward shift during the follow-up period. TOS, total oxidant status; HIFU, high-intensity focused ultrasound.

The distribution of serum TOS values in the HIFU and comparison groups across the three assessment time points is presented in Figure 2.

The distribution of serum TOS values showed a clear downward shift in the HIFU group from baseline to 3 months, followed by a partial upward shift at 6 months. In contrast, the comparison group demonstrated a progressive upward shift throughout the 6-month follow-up period.

**Figure 3:**
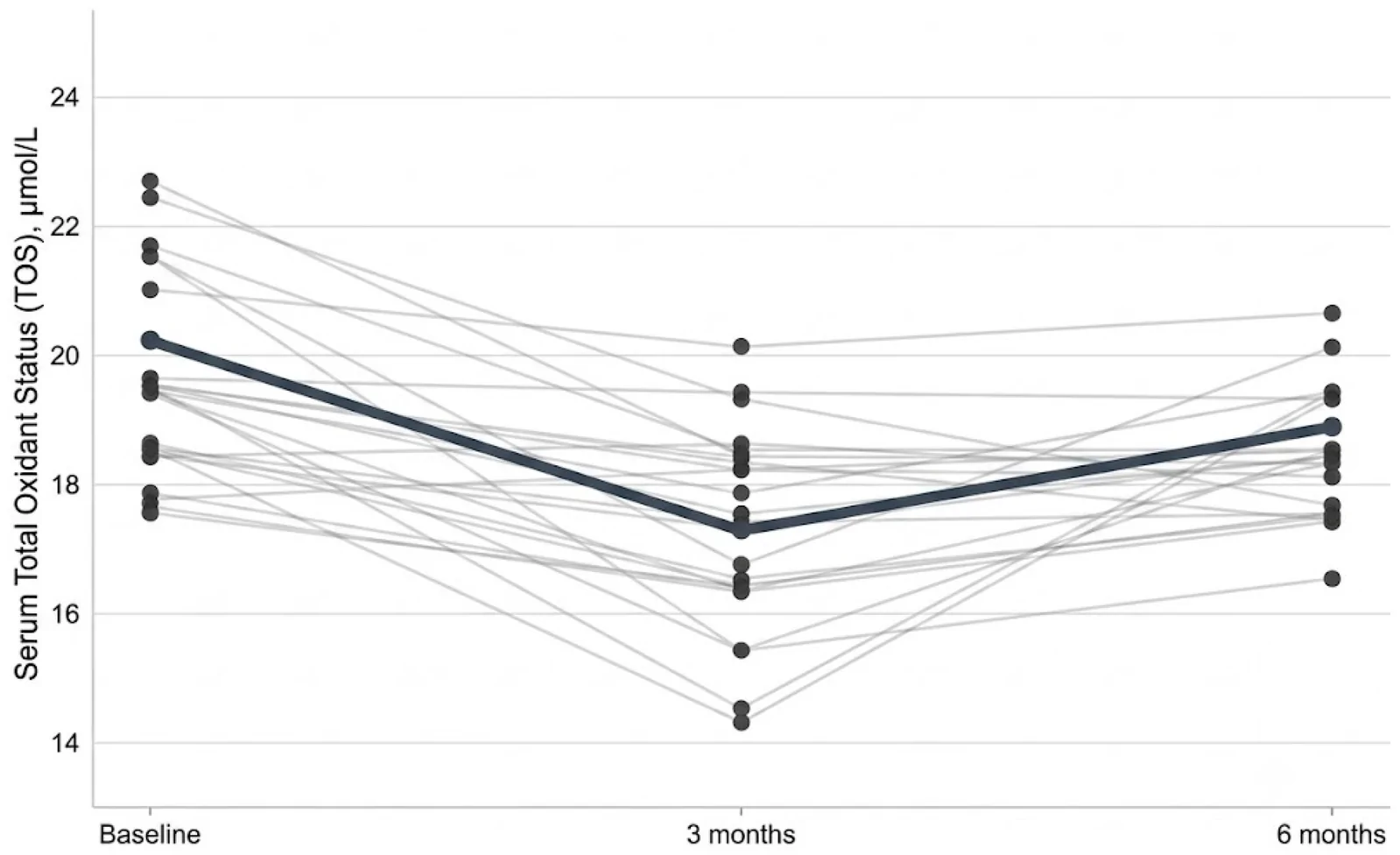
Individual longitudinal trajectories of serum total oxidant status in the HIFU group. Each line represents one participant and connects serum TOS measurements obtained at baseline, 3 months, and 6 months. Most participants demonstrated a reduction in TOS at 3 months, followed by a partial increase at 6 months. TOS, total oxidant status; HIFU, high-intensity focused ultrasound.

Individual participant trajectories are presented in Figure 3. A reduction in TOS from baseline to 3 months was observed in 23 of the 25 participants in the HIFU group. Between 3 and 6 months, TOS increased in 18 participants, reflecting a partial return toward baseline values. Nevertheless, 22 of the 25 participants continued to exhibit lower TOS values at 6 months than at baseline.

### 3.4. Safety, Tolerability, and Adverse Events

No serious, prolonged, or unexpected adverse events were recorded in either study group throughout the 6-month follow-up period. The single facial and neck high-intensity focused ultrasound (HIFU) intervention demonstrated an excellent safety and tolerability profile, with a notable absence of surface-level cutaneous side effects. No participant exhibited post-procedural erythema, edema, blistering, or hyperpigmentation. Regarding tolerability, the vast majority of participants tolerated the procedure well. Mild, transient deep tissue tenderness—described subjectively as an internal bruised sensation—was reported by only three volunteers (12.0%) in the HIFU group. This sensation was mild in intensity, localized to the treated areas, and resolved spontaneously within 3 to 5 days without requiring any medical intervention or analgesia. No cases of peripheral nerve injury or delayed complications were observed during the entire study.

## 4. Discussion

The present prospective controlled pilot study evaluated the longitudinal systemic total oxidant status (TOS) profile following a single facial and neck HIFU treatment. Serum TOS was assessed before treatment (pretreatment baseline) and again at 3 and 6 months. The principal finding was a significant group × time interaction, indicating different longitudinal TOS trajectories between the HIFU and untreated comparison groups. In the HIFU group, TOS decreased from pretreatment baseline to 3 months and remained below baseline at 6 months, despite a partial increase between the two follow-up assessments. In contrast, the comparison group showed a progressive increase over the same period. These findings indicate that, within the present cohort, a single HIFU session was not followed by sustained elevation of systemic TOS during the 6-month observation period.

The 3- and 6-month assessments were selected because these intervals correspond to the period during which HIFU-related collagen remodeling and clinical changes have been reported to develop [1,2,3,4,5,6,13]. HIFU produces localized thermal coagulation within selected tissue planes, followed by tissue-repair and extracellular-matrix remodeling processes [1,2,6,13]. In this context, assessment at pretreatment baseline and at 3 and 6 months allowed for characterization of the systemic redox profile across a clinically relevant follow-up period. However, the observed changes in circulating TOS should not be interpreted as direct evidence that systemic redox alterations reflect or mediate local collagen or extracellular-matrix remodeling, because these tissue-specific processes were not directly assessed in the present analysis.

The current findings also complement our previously published investigation of the acute systemic oxidative response following facial and neck HIFU treatment [14]. In that study, a transient increase in systemic oxidative burden was observed shortly after treatment, whereas the present longitudinal analysis evaluated TOS from pretreatment baseline to 3 and 6 months and demonstrated no sustained elevation during longer-term follow-up. Fourteen HIFU-treated participants contributed to both investigations, while the present cohort included 11 additional HIFU-treated participants. Because the acute and longitudinal responses were evaluated in separate analyses and the cohorts only partially overlapped, the combined findings should not be interpreted as establishing a defined biphasic biological mechanism. Rather, they indicate that the acute oxidative response previously observed was not followed by persistent systemic TOS elevation at 3 or 6 months.

The inclusion of an untreated comparison group provides additional context for interpreting the longitudinal findings. Pretreatment baseline TOS values were comparable between groups, whereas their trajectories subsequently diverged at 3 and 6 months. Both groups followed the same standardized skincare regimen throughout the follow-up period, reducing differential exposure to topical skincare between groups. Nevertheless, group allocation was based on participant preference rather than randomization, and the comparison group was small. Consequently, unmeasured between-group differences and residual confounding cannot be excluded. The findings therefore support an association between HIFU exposure and the observed longitudinal TOS trajectory rather than establishing an exclusive causal effect.

Importantly, the significant group × time interaction indicates that the longitudinal TOS trajectory differed between the two groups, with TOS decreasing in the HIFU group while increasing in the untreated comparison group. This divergence represents a noteworthy finding of the present pilot study, although its biological and clinical significance remains to be established.

The partial increase in TOS between 3 and 6 months in the HIFU group should also be interpreted cautiously. Although TOS increased between these two follow-up assessments, values at 6 months remained below the pretreatment baseline, indicating that the greatest reduction occurred at 3 months followed by a partial movement toward baseline. Participant-level trajectories were generally consistent with this overall pattern. Importantly, a reduction in serum TOS should not itself be interpreted as evidence of a direct antioxidant effect of HIFU. TOS provides an integrated estimate of systemic oxidant burden but does not identify the individual oxidant species involved, their tissue of origin, or the relationship between oxidant production and antioxidant capacity [11]. Serum measurements also cannot determine whether the observed changes originated from the treated tissues or reflected broader systemic influences.

Although the longitudinal and between-group differences in TOS were statistically significant, their biological and clinical significance remains uncertain. A universally accepted age-specific clinical reference interval defining “normal” or “abnormal” serum TOS has not been established for adults in the age range examined in the present study. In the original colorimetric method described by Erel [11], serum TOS was significantly higher in patients with osteoarthritis than in healthy individuals, demonstrating that TOS may vary according to systemic health status. However, these values represent study-specific distributions rather than validated clinical reference intervals. Absolute TOS values may also vary according to the analytical method, assay conditions, pre-analytical factors, and characteristics of the study population. Accordingly, direct numerical comparisons between studies should be interpreted cautiously.

Evidence from other clinical settings further supports the interpretation of TOS as a responsive but nonspecific marker of systemic oxidant burden. In patients with acute appendicitis, TOS was reported to be higher than in healthy controls, with higher levels observed in perforated disease than in less advanced forms of appendicitis [19]. Similarly, in patients with multiple blunt trauma, higher TOS levels were associated with greater injury severity and were observed in nonsurvivors compared with survivors [20]. These observations indicate that circulating TOS can vary in relation to inflammatory and injury-related states. However, they do not establish a single diagnostic, prognostic, or clinically meaningful threshold applicable across different populations or conditions.

Against this broader clinical background, the reduction in TOS observed following HIFU in the present study should not be interpreted as evidence of improved systemic health or as proof of an antioxidant action of HIFU. Rather, the most conservative interpretation is that the HIFU-treated group did not demonstrate sustained elevation of systemic oxidant burden during the 6-month follow-up period. The findings are therefore most appropriately interpreted in terms of longitudinal change from pretreatment baseline to 3 and 6 months and the divergence in TOS trajectories between the HIFU and comparison groups, rather than in relation to a predefined biological or clinical threshold.

No serious treatment-related adverse events were recorded during the 6-month follow-up period. Mild, transient deep-tissue tenderness was the only treatment-related reaction reported and resolved spontaneously. These findings are consistent with published evidence supporting the overall tolerability of HIFU when appropriate treatment parameters and anatomical techniques are used [3,4,5,6,12]. Nevertheless, the absence of serious adverse events in this small pilot cohort should not be interpreted as definitive evidence of long-term safety or the absence of rare adverse events. Similarly, the absence of sustained systemic TOS elevation from pretreatment baseline through the 3- and 6-month assessments indicates that prolonged systemic oxidative burden was not observed in this cohort but does not establish a direct antioxidant effect of HIFU.

### Limitations

Several limitations should be considered when interpreting the present findings. First, the study included a small and unequal sample, particularly the untreated comparison group (HIFU, *n* = 25; comparison, *n* = 5). This limits statistical precision, subgroup evaluation, and the generalizability of the findings. The study was designed as an exploratory pilot investigation rather than a definitive confirmatory trial [15,16,17].

Second, group allocation was based on participant preference rather than randomization. Although pretreatment baseline TOS values were comparable between groups and laboratory personnel responsible for serum processing and biochemical analysis were blinded to group allocation and sampling timepoint, the absence of random assignment introduces the possibility of selection bias and residual confounding. The predominance of female participants and the restricted age range of 45–55 years further limit the extent to which the findings can be generalized to broader populations.

Third, follow-up was limited to 6 months. Consequently, the persistence or further evolution of the TOS trajectory beyond the pretreatment baseline, 3-month, and 6-month assessments remains unknown. Previous HIFU exposure was recorded but was not used as an exclusion criterion, and the sample size was insufficient to determine whether prior exposure may have influenced the longitudinal response.

Fourth, systemic oxidative status was assessed using a single circulating biomarker. Although TOS provides an integrated measure of oxidant burden [11], it does not capture the full complexity of redox homeostasis and cannot characterize antioxidant capacity, specific oxidative-damage products, inflammatory responses, or local tissue-remodeling processes. The present study therefore cannot establish the biological mechanisms underlying the changes observed from pretreatment baseline to 3 and 6 months.

Finally, both groups received the same standardized skincare regimen to minimize differences in topical exposure. However, adherence was assessed by self-report rather than by an objective compliance measure, and the possible contribution of individual skincare ingredients to systemic TOS cannot be completely excluded.

Future studies should include larger and more balanced randomized groups, broader age and sex representation, and longer follow-up periods. Incorporating complementary oxidative, antioxidant, inflammatory, and tissue-remodeling biomarkers together with standardized objective clinical assessments would help determine the biological significance of the TOS trajectory observed from pretreatment baseline through 3 and 6 months and clarify its relationship with local tissue responses and clinical outcomes following HIFU treatment.

## 5. Conclusions

This prospective controlled pilot study demonstrated a distinct longitudinal pattern of serum total oxidant status following a single facial and neck HIFU treatment. Serum TOS decreased significantly from the pretreatment baseline to 3 months and remained significantly below baseline at 6 months, despite a partial increase between the two follow-up assessments. In contrast, the untreated comparison group showed a progressive increase in TOS over the same period, resulting in a significant group × time interaction. These findings indicate that the longitudinal systemic redox profile differed between HIFU-treated and untreated participants during the 6-month follow-up period.

Within the limitations of this pilot study, a single HIFU session was not associated with sustained elevation of systemic TOS during follow-up, and no serious treatment-related adverse events were observed. However, the observed reduction in TOS should not be interpreted as evidence of a direct antioxidant effect of HIFU, as TOS represents an integrated systemic measure and does not identify specific molecular mechanisms or tissue sources. Larger randomized studies with more balanced comparison groups, longer follow-up, and complementary oxidative, antioxidant, inflammatory, and tissue-remodeling biomarkers are required to confirm the present findings and further clarify their biological significance.

## Figures and Tables

**Table 1:** Baseline Demographic, Lifestyle, and Clinical Characteristics of the Study Participants.

Characteristic	HIFU Group (*n* = 25)	Comparison Group (*n* = 5)	Total (*n* = 30)
Age, years, mean ± SD	49.24 ± 3.99	51.40 ± 5.41	49.60 ± 4.23
Age range, years	45–55	45–55	45–55
Women, *n* (%)	24 (96.0)	4 (80.0)	28 (93.3)
Men, *n* (%)	1 (4.0)	1 (20.0)	2 (6.7)
Current smokers, *n* (%)	13 (52.0)	3 (60.0)	16 (53.3)
Self-reported stress score, mean ± SD ^1^	7.68 ± 1.70	6.00 ± 2.35	7.40 ± 1.89
No regular physical activity, *n* (%)	8 (32.0)	1 (20.0)	9 (30.0)
Light physical activity, *n* (%)	11 (44.0)	3 (60.0)	14 (46.7)
Regular physical activity, *n* (%)	6 (24.0)	1 (20.0)	7 (23.3)
Regular sunscreen use, *n* (%)	20 (80.0)	4 (80.0)	24 (80.0)
Chronic medication use, *n* (%)	9 (36.0)	3 (60.0)	12 (40.0)
Standardized skin-care regimen, *n* (%)	25 (100)	5 (100)	30 (100)

Data are presented as mean ± standard deviation (SD), range, or number (percentage), as appropriate. ^1^ Self-reported stress was assessed using a numerical scale ranging from 1 to 10 and was analyzed descriptively.

**Table 2:** Observed and Model-Estimated Serum Total Oxidant Status at Baseline and at the 3- and 6-Month Follow-Up Assessments.

Variable	HIFU Group (*n* = 25)	Comparison Group (*n* = 5)
Baseline TOS (μmol/L), mean ± SD	19.54 ± 1.49	19.78 ± 1.81
3-month TOS (μmol/L), mean ± SD	17.31 ± 1.51	20.55 ± 1.92
6-month TOS (μmol/L), mean ± SD	18.34 ± 0.95	21.61 ± 2.45
Change: Baseline to 3 months (μmol/L), mean ± SD	−2.23 ± 1.71	+0.77 ± 0.58
Change: Baseline to 6 months (μmol/L), mean ± SD	−1.21 ± 1.44	+1.83 ± 1.32
Change: 3 to 6 months (μmol/L), mean ± SD	+1.03 ± 1.63	+1.06 ± 0.90

Values are presented as observed mean ± standard deviation. Changes were calculated as the later measurement minus the earlier measurement; therefore, negative values indicate a reduction in serum TOS. Abbreviations: TOS, total oxidant status; SD, standard deviation; HIFU, high-intensity focused ultrasound.

## Data Availability

The aggregated data supporting the findings of this study are presented within the article. Additional de-identified data may be made available by the corresponding author upon reasonable request, subject to ethical approval and applicable data-protection restrictions. Participant names and other personally identifiable information are not available because of privacy and confidentiality requirements.

## Abbreviations

The following abbreviations are used in this manuscript:

HIFU	High Intensity Focused Ultrasound
TOS	Total Oxidant Status
ELISA	Enzyme-Linked Immunosorbent Assay
SD	Standard Deviation
CI	Confidence Interval
QC	Quality Control
CV	Coefficient of Variation
Within-run CV	Between-run Quality Control
Between-run QC	Change (immediately after HIFU- after 3 months) or (3 months-post HIFU–6 months post-HIFU)
LOQ	Limits of Qualification
Δ	Change (calculated as the later measurement minus the earlier measurement; baseline to 3 months, baseline to 6 months, or 3 to 6 months)
